# Diagnosis of Alzheimer’s disease: recommendations of the Scientific Department of Cognitive Neurology and Aging of the Brazilian Academy of Neurology

**DOI:** 10.1590/1980-5764-DN-2022-S102PT

**Published:** 2022-11-28

**Authors:** Lucas Porcello Schilling, Marcio Luiz Figueredo Balthazar, Márcia Radanovic, Orestes Vicente Forlenza, Marcela Lima Silagi, Jerusa Smid, Breno José Alencar Pires Barbosa, Norberto Anízio Ferreira Frota, Leonardo Cruz de Souza, Francisco Assis Carvalho Vale, Paulo Caramelli, Paulo Henrique Ferreira Bertolucci, Márcia Lorena Fagundes Chaves, Sonia Maria Dozzi Brucki, Benito Pereira Damasceno, Ricardo Nitrini

**Affiliations:** 1 Pontifícia Universidade do Rio Grande do Sul, Escola de Medicina, Serviço de Neurologia, Porto Alegre RS, Brasil.; 2 Pontifícia Universidade do Rio Grande do Sul, Instituto do Cérebro do Rio Grande do Sul, Porto Alegre RS, Brasil.; 3 Pontifícia Universidade do Rio Grande do Sul, Programa de Pós-Graduação em Gerontologia Biomédica, Porto Alegre RS, Brasil.; 4 Universidade Estadual de Campinas, Faculdade de Ciências Médicas, Departamento de Neurologia, Campinas SP, Brasil.; 5 Universidade de São Paulo, Faculdade de Medicina, Hospital das Clínicas, Instituto de Psiquiatria, Laboratório de Neurociências, São Paulo SP, Brasil.; 6 Universidade de São Paulo, Faculdade de Medicina, Departamento de Psiquiatria, São Paulo SP, Brasil.; 7 Universidade Federal de São Paulo, Departamento de Fonoaudiologia, São Paulo SP, Brasil.; 8 Universidade de São Paulo, Faculdade de Medicina, Departamento de Neurologia, Grupo de Neurologia Cognitiva e do Comportamento, São Paulo SP, Brasil.; 9 Universidade Federal de Pernambuco, Centro de Ciências Médicas, Área Acadêmica de Neuropsiquiatria, Recife PE, Brasil.; 10 Instituto de Medicina Integral Prof. Fernando Figueira, Recife PE, Brasil.; 11 Hospital Geral de Fortaleza, Serviço de Neurologia, Fortaleza CE, Brasil.; 12 Universidade de Fortaleza, Fortaleza CE, Brasil.; 13 Universidade Federal de Minas Gerais, Departamento de Clínica Médica, Belo Horizonte MG, Brasil.; 14 Universidade Federal de São Carlos, Centro de Ciências Biológicas e da Saúde, Departamento de Medicina, São Carlos SP, Brasil.; 15 Universidade Federal de São Paulo, Escola Paulista de Medicina, Departamento de Neurologia e Neurocirurgia, São Paulo SP, Brasil.; 16 Hospital de Clínicas de Porto Alegre, Serviço de Neurologia, Porto Alegre RS, Brasil.; 17 Universidade Federal do Rio Grande do Sul, Faculdade de Medicina, Departamento de Medicina Interna, Porto Alegre RS, Brasil.

**Keywords:** Alzheimer Disease, Dementia, Diagnosis, Doença de Alzheimer, Demência, Diagnóstico

## Abstract

This paper presents the consensus of the Scientific Department of Cognitive Neurology and Aging from the Brazilian Academy of Neurology on the diagnostic criteria for Alzheimer’s disease (AD) in Brazil. The authors conducted a literature review regarding clinical and research criteria for AD diagnosis and proposed protocols for use at primary, secondary, and tertiary care levels. Within this clinical scenario, the diagnostic criteria for typical and atypical AD are presented as well as clinical, cognitive, and functional assessment tools and complementary propaedeutics with laboratory and neuroimaging tests. The use of biomarkers is also discussed for both clinical diagnosis (in specific conditions) and research.

## RESUMO

Este artigo apresenta o consenso realizado pelo Departamento Científico de Neurologia Cognitiva e do Envelhecimento da Academia Brasileira de Neurologia sobre os critérios diagnósticos da Doença de Alzheimer (DA) no Brasil. Foi realizada uma revisão da literatura e dos critérios clínicos e de pesquisa para DA, sendo propostos protocolos para o diagnóstico de DA em níveis de atenção primária, secundária e terciária. Dentro deste cenário clínico, são apresentados os critérios diagnósticos para DA típica e atípica, além de instrumentos de avaliação clínica, cognitiva e funcional; bem como propedêutica complementar com exames laboratoriais e de neuroimagem. A utilização de biomarcadores é também apresentada, tanto para o diagnóstico clínico em situações específicas quanto para pesquisa.

## INTRODUCTION

### Epidemiology and relevance

The continuous aging of the world population increases the prevalence and incidence of chronic and neurodegenerative diseases. Currently, dementia affects an estimated 50 million people worldwide and has 10 million new diagnoses per year, of which about 60% are due to Alzheimer’s disease (AD). Projections indicate an estimated 150 million people with dementia due to AD by 2050^1^. In Brazil, an estimated 1.7 million people have dementia, with a prevalence of approximately 1,036/100,000 inhabitants^2^.

#### Risk factors

Risk factors for AD can be divided into environmental and genetic. Environmental factors are more related to the sporadic form of the disease (late-onset or senile AD), whose main risk factor is aging^3^. Other risk factors include low schooling level, systemic arterial hypertension, diabetes, obesity, sedentary lifestyle, head trauma, depression, smoking, hearing loss, and social isolation^4^, which can all be prevented and modified.

From the genetic point of view, mutations responsible for the autosomal dominant forms of AD stand out. Unlike the multifactorial etiology of late-onset sporadic AD, autosomal dominant forms (which are relatively rare) have early onset, occurring before 65 years (presenile AD), and are strongly associated with mutations of the amyloid precursor protein (APP), presenilin 1, or presenilin 2 genes, which are identified in 70% of cases, and with a dominant, autosomal inheritance pattern^5^.

Though late-onset forms of AD are rarely associated with dominant inheritance, they can be related to genetic risk factors such as the ε4 allele of the apolipoprotein E (APOE) gene, which increases the risk for AD development and anticipates its onset in a few years. Homozygosis for the APOEε4 allele increases this risk in five times compared to heterozygotes^6^.

### Pathophysiology

The main hypothesis in AD pathophysiology establishes that the degenerative process is triggered by a hyperproduction and/or decreased clearance and consequent accumulation of amyloid-beta peptide (Aβ) and tau protein in the affected brain tissues, accompanied by homeostatic changes that lead to a collapse of the neuronal cytoskeleton. The APP is usually cleaved by the enzyme α-secretase (ADAM10), generating soluble peptides (APPs); in AD, an alternative and sequential cleavage of APP occurs by secretases β (BACE-1) and y, generating insoluble Aβ peptides that aggregate and deposit in the extracellular space, triggering several pathological events that cause neuronal death and formation of senile or neuritic plaques (NPs). Neurofibrillary tangles (NFTs) are intracellular deposits composed of hyperphosphorylated tau protein. The tau protein maintains the integrity of intraneuronal microtubules, a function that is lost after the hyperphosphorylation process.

The initial clinical symptoms of amnestic AD are related to the increased density of NFTs in the hippocampal formation, nucleus basalis of Meynert, and paralimbic regions (fusiform gyrus and inferior and middle temporal gyri), corresponding to stages III and IV of Braak’s neuropathological staging^7^. In preclinical stages of AD (Braak stages I and II), NFTs occur almost exclusively in limbic system structures (entorhinal cortex, subiculum, and hippocampus, in addition to the amygdala, nucleus basalis of Meynert, and temporopolar cortex). In mild AD (Braak stage V), the density of NFTs increases in the limbic system and they emerge at the associative neocortical regions of the middle and superior temporal gyri (related to language symptoms) as well as at the prefrontal, retrosplenial, and posterior parietal cortices (related to executive dysfunction and spatial disorientation). NPs also deposit in these areas, progressively increasing the density of NFTs and NPs in the whole neocortex, including unimodal (visual, auditory, and somestesic) and multimodal association areas in the temporoparietal and dorsolateral frontal junction. Severe AD corresponds to Braak advanced stage VI, in which all cortical association areas and the basal ganglia are affected by NFTs and NPs, with relative sparing of the motor and sensory cortices.

Other pathophysiological mechanisms in AD include synaptic dysfunction, neurotransmitters (mainly acetylcholine) and neurotrophin depletion, mitochondrial dysfunction and deficits in insulin signaling pathways, increase in oxidative stress and inflammation, vascular changes^8^, and cholesterol metabolism^9^. Recent studies suggest that the interaction between different pathophysiological processes, such as white matter involvement associated with Aβ accumulation^10^ and soluble Aβ oligomers interaction with other proteins (such as α-synuclein and tau), destabilize microtubules, mitochondrial and synaptic dysfunction, and neurodegeneration^11^ ^),(^ ^12^. 

## CLINICAL CHARACTERIZATION

### Clinical characterization

AD usually manifests in the typical amnestic presentation, with a predominant difficulty of episodic memory associated with degenerative lesions of medial temporal structures. This pattern occurs in about 85% of cases. Atypical and less frequent presentations may begin mainly with impairment in language, visual-spatial, executive, or complex motor functions. The most common atypical (usually presenile) forms are the logopenic variant of primary progressive aphasia (lvPPA) and the visual-spatial-apraxic of posterior cortical atrophy (PCA), whereas the least common forms are the corticobasal syndrome (CBS) and the behavioral and dysexecutive variant (ADbdv).

Due to these non-amnestic variants, memory impairment is no longer mandatory for AD diagnosis according to the most recent diagnostic criteria from the Brazilian Academy of Neurology^13^ based on the National Institute on Aging-Alzheimer’s Association (NIA-AA) ^(^ ^14^. To help identify these initial forms of AD presentation, we will detail their clinical characteristics, evolution stages, and corresponding neuropathological substrates.

#### AD amnestic presentation

The typical AD presentation starts with difficulties remembering messages and recent news and repeating the same questions, comments, and narratives. Initially mild and intermittent, the symptoms first appear as subjective memory impairment (SCI) followed by mild cognitive impairment (MCI) - usually of the multiple-domain amnestic type (also impairing the language and executive functions) -, later evolving to full-blown dementia, when they begin to interfere in the activities of daily living and in the patient’s autonomy^14^.

#### Logopenic variant of primary progressive aphasia (lvPPA) 

The first and predominant symptoms in this variant are language alterations with non-fluent speech, pauses due to word-finding difficulty, and errors (also phonological) when repeating long sentences and in spontaneous speech, but preserving semantics, syntax (grammar), comprehension of single words, and motor production of speech^15^ ^),(^ ^16^.

#### Posterior cortical atrophy (PCA) or posterior variant

PCA is a rare form of AD which usually starts between the ages of 50 and 60 years. Its occipitotemporal variant presents with impairment of the visual identification of objects, faces, or symbols; its biparietal variant, more common, is characterized by visual-spatial dysfunction, topographical disorientation, poor hand-eye coordination, limb apraxia, visual neglect, and at clinical evaluation, elements of Balint’s syndrome (optic ataxia, oculomotor apraxia, and simultanagnosia) and/or Gerstmann syndrome (acalculia, agraphia, left-right disorientation, and finger agnosia) ^(^ ^17^ ^),(^ ^18^.

In the early stages of the disease, episodic memory, language, and executive functions are still relatively preserved. PCA presents with AD neuropathology in 62 to 100% of cases^19^.

#### Behavioral and dysexecutive variant (ADbdv)

The AD dysexecutive variant (ADdv) affects mainly planning, working memory, and multi-tasking, with loss of inhibitory control and alternating attention, depression, anxiety and neuropsychiatric symptons. In turn, behavioural symptoms are rare. 

Though the behavioral variant of AD is similar to the behavioral variant of frontotemporal dementia (bvFTD), it presents greater deficits in memory, apathy, delusional ideas, and hallucinations, with less disinhibition, compulsive or persevering behavior, affective indifference, or personality change^20^ ^)-(^ ^23^. This presentation of AD is rare, occurring in about 2% of large samples of AD patients and with 7-20% of patients clinically diagnosed as FTD^19^ ^),(^ ^24^ ^),(^ ^25^.

#### Corticobasal syndrome (CBS)

CBS manifests with remarkably asymmetric or unilateral signs and symptoms of stiffness, dystonia, myoclonus, bradykinesia, and tremor. It is associated with gait alteration, asymmetric apraxia, alien hand phenomenon, sensory hemineglect, and visual-spatial deficits, besides the more typical symptoms of episodic and visual-spatial memory deficits and aphasia^19^ ^), (^ ^23^. AD causes 15-50% of CBS cases, degenerating cortical structures and basal ganglia, including the substantia nigra, and manifesting clinically mainly as motor symptoms.

### Clinical stages of dementia

#### Mild dementia

The mild dementia stage is characterized by progressive worsening of amnestic symptoms associated with other cognitive disorders, such as impaired working memory, attentional control (difficulty multitasking), language alterations (anomia), executive dysfunction (struggles with planning, problem-solving), and temporal-spatial disorientation^26^.

Neuropsychiatric symptoms occur in all stages (in up to 80% of cases) and worsen as dementia progresses, especially apathy, depression, anxiety^27^ ^),(^ ^28^, and a lack of awareness regarding cognitive deficits (anosognosia), which occurs in up to 50% of patients^19^ ^),(^ ^29^.

#### Moderate dementia

In the moderate dementia stage, patients become more dependent on others to perform instrumental activities of daily living (although still capable of self-care) and have greater difficulty remembering the name of relatives, remote events, or more significant recent events. Other cognitive symptoms may worsen, such as temporal and spatial disorientation, development of transcortical sensory aphasia, ideomotor apraxia, dyscalculia, visual agnosia, and neuropsychiatric symptoms such as delusions (typically of betrayal or theft), hallucinations, and agitation, with or without aggressiveness.

#### Severe dementia

In the severe dementia stage, patients are entirely dependent on a caregiver, with memory reduced to fragments of information, temporal and personal disorientation (maintaining only self-awareness), and speech restricted to a few unintelligible words. In more advanced stages, they can present urinary and fecal incontinence, parkinsonism, myoclonus, epileptic seizures (in up to 20% of cases) ^(^ ^30^ ^),(^ ^31^, and gait difficulties. Later, patients have difficulty sitting and swallowing. The average survival time is five to 12 years after the onset of symptoms, but with significant variability among patients^32^.

### Diagnosis

#### Clinical diagnosis of AD

The clinical diagnosis of AD dementia is based on a thorough evaluation, especially of the patient’s affected cognitive domains and functional impairment, as described in the diagnostic criteria and neuropsychological assesment section^13^ ^),(^ ^14^. AD is a progressive pathological process with different clinical stages, and dementia occurs when pathological changes have already spread^33^.

Understanding this cognitive continuum is essential for an appropriate clinical evaluation of the patient and, with complementary tests (including biomarkers), an accurate diagnosis in atypical or early-onset cases. Biomarkers also allow identifying patients and indicating possible future specific treatments for AD^34^.

Diagnosis of dementia due to Alzheimer’s disease (modified from McKhann et al., 2011^13^ and Frota et al., 2011^14^

- Probable Alzheimer’s disease dementia

If the patient meets the criteria for diagnosis of dementia^35^ and has the following characteristics:

I. Insidious onset (months or years);
II. Clear history or observation of cognitive worsening;
III. Initial and more prominent cognitive deficits in one of the following categories:
•Amnestic presentation (there must be another affected domain).
•Non-amnestic presentation (there must be another domain affected).
•Language (memory of words).
•Visual-spatial (spatial cognition or agnosia for objects or faces, simultanagnosia, and alexia).
•Executive functions (alterations of reasoning, judgment, and problem-solving).
IV. Tomography or, preferably, magnetic resonance imaging of the brain should be performed to exclude other diagnostic possibilities or comorbidities, especially cerebrovascular disease;
V. The diagnosis of probable AD dementia should not be applied when there are:
•Evidence of significant cerebrovascular disease defined by a history of stroke temporally related to the onset or worsening of cognitive impairment; presence of multiple or extensive brain infarctions; or extensive lesions in the white matter evidenced by neuroimaging; or
•Central features of dementia with Lewy bodies (visual hallucinations, parkinsonism, REM sleep behavior disorder, and cognitive fluctuation); or
•Prominent features of the behavioral variant of FTD (hyperorality, hypersexuality, perseveration); or
•Prominent characteristics of PPA manifesting as semantic variant (with fluent speech, anomia, and semantic memory difficulties) or as non-fluent variant (with agrammatism and/or marked speech apraxia); or
•Evidence of concomitant neurological or non-neurological active disease, or medication use that may substantially affect cognition.

#### Anamnesis

A detailed anamnesis focused on the most common cognitive and neuropsychiatric alterations of AD allows diagnosing the disease more safely, establishing its subtype based on its initial presentation and stage, and differentiating it from other neurodegenerative diseases. Interrogation of patients and their relative/caregiver should cover (1) neuropsychiatric disorders such as depression, anxiety, apathy, delusional ideas, hallucinations, and aberrant or uninhibited motor behaviors, which are socially inappropriate; and (2) cognitive difficulties in the following domains, most affected by the disease:

- Episodic memory: Does the patient forget recent facts and dates, items to purchase, appointments, or places where he/she keeps objects? Or does he/she keep repeating the same questions or comments?
- Executive functions: Does the patient have difficulty staying focused, making decisions, planning activities, solving everyday problems, shopping, and dealing with small amounts of money? Do they present loss of motivation and initiative? Do they have impaired judgment?
- Visual-spatial or praxic skills: Does the patient have difficulty orienting themselves spatially (outside and indoors), dressing, combing, shaving, using everyday objects, recognizing familiar faces? Have they lost dexterity in tasks in which they used to do well?
- Language: Does the patient have difficulty finding words in conversations or naming objects and people? Or in understanding words or sentences, explaining situations and making themselves understood, presenting poor vocabulary and reduced speech fluency?

#### Neuropsychological assessment

According to specific studies on the subject^36^ ^),(^ ^37^, the diagnosis of AD in its initial stage (or MCI) has greater reliability when using two tests for each of the four cognitive domains most affected by the disease and greater sensitivity when defining deficit score as >1 standard deviation (SD), and not >1.5 or >2SD), relative to normative values. Thus, in addition to conducting a global cognitive test (MMSE, Mini-Mental State Examination; or MoCA, Montreal Cognitive Assessment), the evaluation must include episodic memory, language, executive functions, and visual-spatial functions, with two subtests for each cognitive domain.

The main instruments recommended for cognitive assessment in AD in Brazil are presented below. Given the country’s socio-cultural and educational heterogeneity, it is advisable to use instruments with cutoff scores adjustable by level of education to avoid false-positive results in the diagnostic process^38^. The instruments are subdivided into cognitive screening tests, specific tests for evaluating different cognitive domains, and instruments for assessing functionality (Table 1).

**Table 1:** Main instruments for cognitive assessment in AD.

Type of instrument	Main tests and normative studies
Screening tests	
Brief tests	Mini-Mental State Examination (MMSE) ^(^ ^39^ ^),(^ ^40^, Montreal Cognitive Assessment (MoCA) ^(^ ^41^ ^),(^ ^42^, Cognitive Abilities Screening Instrument - Short Version (CASI-S) ^(^ ^43^ ^),(^ ^44^, Brief Cognitive Screening Battery (BBRC) ^(^ ^45^ ^),(^ ^46^
Multi-functional batteries	Addenbrooke’s Cognitive Examination - revised version (ACE-R) ^(^ ^47^ ^),(^ ^48^, Cambridge Cognitive Examination (CAMCOG) ^(^ ^49^ ^)-(^ ^51^, Alzheimer’s Disease Assessment Scale-Cognitive Subscale (ADAS-COG) ^(^ ^52^ ^),(^ ^53^, Consortium to Establish a Registry for Alzheimer’s Disease (CERAD) ^(^ ^54^ ^),(^ ^55^, Mattis Dementia Rating Scale (MDRS) ^(^ ^56^ ^),(^ ^57^
**Evaluation of single cognitive domains**	
Verbal episodic memory	Rey Auditory Verbal Learning Test (RAVLT) ^(^ ^58^ ^),(^ ^59^, CERAD Battery Word List Learning Subtest
Nonverbal memory	Subtest “figure recognition” (BBRC), Subtest “geometric figure recall” (CERAD Battery), Rey-Osterrieth Complex Figure^60^ ^),(^ ^61^
Language	Verbal Fluency Test (phonemic and semantic) ^(^ ^62^ ^),(^ ^63^, Boston Naming Test (BNT) ^(^ ^64^ ^),(^ ^65^
Attention control and executive function	Digit Span Task in forward and inverse order^66^ ^),(^ ^67^, Clock Drawing Test^68^ ^),(^ ^69^, Verbal Fluency Test
Visual-spatial / visual-constructive abilities	CERAD / MoCA Figure Copy Subtest, Clock Drawing Test
**Functional assessment**	
Instrumental activities of daily living	Functional Activities Questionnaire (FAQ) ^(^ ^70^ ^),(^ ^71^, Informant Questionnaire on Cognitive Decline in the Elderly (IQCODE) ^(^ ^72^ ^),(^ ^73^, Direct Assessment of Functional Status-Revised (DAFS-R) ^(^ ^74^ ^),(^ ^75^, Disability Assessment for Dementia (DAD) ^(^ ^76^ ^),(^ ^77^, Activities of Daily Living Questionnaire (ADLQ) ^(^ ^78^ ^),(^ ^79^, Bayer Activities of Daily Living Scale (B-ADL) ^(^ ^80^ ^),(^ ^81^, AD8 Dementia Screening Interview^82^
Basic activities of daily living	Katz scale^83^ ^),(^ ^84^, Functional Activities Questionnaire (FAQ) ^(^ ^70^ ^),(^ ^72^
Dementia staging	Clinical Dementia Rating scale (CDR) ^(^ ^85^ ^),(^ ^86^

Note: The global CDR scores (CDR-GS: 0, 0.5, 1, 2, or 3) have the limitation of being based on the scores of the item Memory, considering the other items as secondary, and thus underestimating relevant information from instrumental activities that may be primarily and early affected.

More recently, the sum of boxes (CDR-Sum of Boxes - CDR-SB) have replaced the Clinical Dementia Rating (CDR) global scores. The first allows detecting smaller differences within and between subsequent global scores as well as within and between stages of the disease, helping differentiate MCI from initial dementia, as seen in O’Bryant et al. ^(^ ^87^, who described the following ranges of CDR-SB corresponding to CDR-GS scores: 0.5 to 4.0 for the CDR-GS of 0.5; 4.5 to 9.0 for CDR-GS of 1; 9.5 to 15.5 for CDR-GS of 2; and 16.0 to 18.0 for CDR-GS of 3.

#### Laboratory tests in AD clinical propaedeutics

Several clinical conditions may cause cognitive impairment, such as hypothyroidism, hypovitaminosis, and neurosyphilis. An initial medical evaluation should conduct a basic laboratory evaluation to rule out the main secondary causes of cognitive decline. It should also seek to identify systemic diseases and comorbidities that could worsen the neurological condition, such as dyslipidemia and diabetes^88^ ^)-(^ ^91^.

The list of recommended laboratory tests should include hematological, renal, hepatic, lipid, and metabolic profiles (serum sodium, potassium, and calcium), fasting glucose, folic acid dosage, vitamin B12, TSH, free T4, syphilis serology, and especially in atypical cases or in case of clinical suspicion, HIV testing^92^.

#### Structural neuroimaging

Brain evaluation by structural neuroimaging such as computed tomography (CT) or magnetic resonance imaging (MRI) is essential for properly diagnosing AD, both to rule out secondary lesions and to identify patterns of brain atrophy specific to the disease. MRI provides better anatomical resolution and different acquisition techniques that are more useful than CT for differential diagnoses with other dementias, such as those of vascular or prion pathology.

As a neurodegenerative disease, AD invariably occurs with cerebral atrophy. The most common pattern of volumetric alteration is atrophy of mesial temporal structures (MTS), in structures such as the hippocampus and entorhinal cortex, which correlates with the clinical findings of episodic memory deficit. However, atrophy can also affect different regions, especially in atypical presenile presentations, such as linguistic, dysexecutive and/or behavioral (frontal), and visual-spatial variants, among others, which will be discussed later^93^.

**Figure 1:**
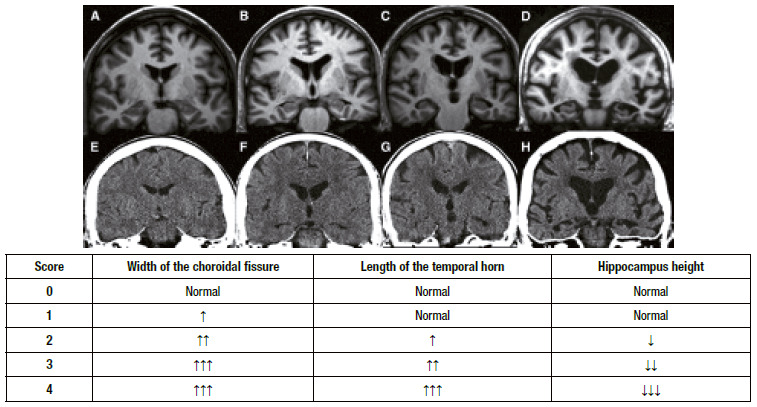
Application of the MTA scale on MRI (above) and CT (below). Under 75 years old, ≥2 is abnormal; over 75 years old, ≥3 is abnormal^95^.

**Figure 2:**
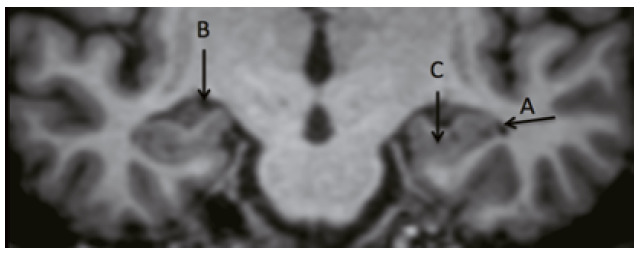
Structures evaluated in the MTA scale. A: temporal horn; B: choroidal fissure; C: hippocampus.

Computed tomography (CT) is a useful, more available, and lower-cost effective study that can be used even in primary health care. Similarly to MRI, CT can rule out structural lesions such as subdural hematoma, tumors, and hydrocephalus^94^. It can also evaluate hippocampal atrophy, especially via coronal plane reconstruction. Although MRI has a higher anatomical resolution than CT, the Medial Temporal Atrophy Scale (MTA or Scheltens scale) can also be used in CT (Figure 1) ^(^ ^95^. The MTA is sensitive to diagnose AD and specific to differentiate AD from normal older adults, although other dementias may also present hippocampal atrophy, such as vascular dementia or dementia with Lewy bodies. The Scheltens scale assesses the width of the choroidal fissure and temporal horn as well as the height of the hippocampus(Figure 2) ^(^ ^94^ ^),(^ ^96^. 

As aforementioned, MRI presents better anatomical resolution and allows performing other imaging techniques. Visual inspection of MTS using scales such as the MTA is still the most widespread and available method in the clinical diagnosis of AD, with sensitivity and specificity of 80 to 85% in differentiating individuals with AD from cognitively normal individuals^97^. Some radiological centers and software available on the Internet measure hippocampal volume by MRI, which may increase sensitivity/specificity for diagnosis. Although this information is essential, especially for follow-up, it still lacks standardization for the Brazilian population considering age and sex. Regarding MTS in AD, the subregions of the hippocampus, such as CA1 and subiculum, can be quantified, but without widespread clinical application^98^. A recent review of MCI due to AD showed low sensitivity/specificity (73 and 71%, respectively) of MTS measures to differentiate patients whose condition evolved or not to dementia^99^.

Other MRI techniques do not yet have a consolidated role in clinical practice for AD diagnosis. As an example, although the MRI proton magnetic resonance spectroscopy technique - which assesses brain metabolites such as N-acetylaspartate (Naa), creatine (Cr), and myo-inositol (mI) - shows differences between groups of patients with and without AD, it still has many limitations and technique heterogeneity as to be applied in clinical practice as a marker of the disease. The main findings of the technique are a decrease in NAA and NAA/Cr ratio and an increase in mI and mI/Cr ratio^100^.

Several other techniques, such as diffusion tensor imaging, texture analysis, MRI infusion, and functional connectivity, are still restricted for research.

#### Structural neuroimaging in atypical AD

A common feature of atypical presentations of AD, is the relative preservation of MTS in relation to atrophy of other brain regions. Each variant has its imaging characteristics, which generally correlate with clinical symptoms. Some neuroimaging characteristics of the three most common atypical presentations will be briefly described: visual-spatial variant (part of the PCA spectrum); linguistic variant, most commonly lvPPA; and ADbdv.

- PCA: predominance of parietal and posterior temporal atrophy. The Koedam’s parietal atrophy scale ranges from 0 to 3 and assesses the integrity of the precuneus and dilatation of the posterior cingulate, parieto-occipital, and parietal lobe sulci. It may be helpful in the diagnosis of PCA, and scores ≥ 2 can be considered abnormal, according to the author’s proposal^101^;
- lvPPA: asymmetric atrophy of temporoparietal structures, predominantly in the left hemisphere (dominant for language) ^(^ ^16^;
- ADbdv: is the most heterogeneous presentation in terms of image. It presents greater atrophy in the dorsolateral prefrontal cortex compared to typical AD but may also present a pattern of temporoparietal atrophy^20^.

### Biomarker-assisted diagnosis

#### Biomarkers in cerebrospinal fluid

The cerebrospinal fluid (CSF) biomarkers used for AD diagnosis are the 42-amino acid Aβ peptide (Aβ1-42) and the tau protein in its total composition and as phosphorylated residue at threonine 181 (T-tau and P-tau, respectively). The “AD pathological signature” in CSF consists of a pattern determined by reduced Aβ1-42 concentration and increased concentrations of T-tau and P-tau^102^ ^)-(^ ^104^.

In recent years, the use of CSF AD biomarkers for diagnosis has significantly advanced. Ideally, concentrations of Aβ1-42 should be normalized in relation to those of Aβ1-40, which do not vary significantly among dementias. The ratio Aβ1-42/Aβ1-40 can also better predict the PET measurement of amyloid load than the concentration of Aβ1-42 alone^105^ ^)-(^ ^107^.

There is a correspondence between the pattern of CSF biomarkers and the pathophysiological changes underlying AD. The reduction of Aβ1-42 and the increase of P-tau in CSF indicate cerebral amyloidosis and tauopathy, mechanisms that form, respectively, NPs and NFTs. The increase in T-tau signals the ongoing neurodegenerative process, usually represented by structural changes (atrophy) and regional metabolic impairment.

#### Molecular neuroimaging biomarkers

Pathophysiological processes related to AD can be alternatively inferred in vivo by molecular imaging methods based on positron emission tomography (PET) by injecting different radiotracers.

The progressive degenerative process of AD causes cerebral hypometabolism, which can be assessed using [18F]Fluorodeoxyglucose (FDG) as a tracer. Patients with AD present hypometabolism patterns involving the posterior cingulate, precuneus, temporoparietal, and medial temporal cortices^108^ ^)-(^ ^111^.

Several molecular agents can evaluate the cerebral accumulation of Aβ from peptide Aβ affinity, such as the [11C]Pittsburgh compound-B (PiB), the [18F]Flutemetamol, the [18F]Florbetaben, and [18F]Florbetapir^112^ ^),(^ ^113^. PiB presents a greater limitation to clinical use since it has carbon in its molecular structure, with a half-life of only 20 minutes. Compared to the measurement of Aβ in CSF, molecular imaging by PET has the advantage of topographically identifying Aβ accumulation in the brain, involving the precuneus and bilateral fronto-temporo-parietal cortices^113^.

PET-specific radiotracers, such as [18F]Flortaucipir, can also assess the accumulation of tau protein, an essential pathological characteristic of AD ^114^. Tau accumulation observed in molecular neuroimaging studies has significant clinical correlations: a greater accumulation of tau is related to the increased severity of cognitive decline^115^ ^)-(^ ^117^ ^)^ and, in atypical AD, the regions with higher radiotracer retention are associated with symptoms related to these regions, such as occipital lobes in PCA, and to the pattern of glycolytic hypometabolism^118^. Aβ biomarkers have good sensitivity to identify cases of incipient AD^119^ whereas P-Tau markers have greater specificity to diagnose AD^120^ ^),(^ ^121^.

Currently, biomarkers are used mainly in research. From the clinical point of view, biomarkers should be used to assess conditions considered atypical, either for an initial non-amnestic clinical presentation or in patients with early-onset dementia, which will be further addressed in the next section.

#### When to request CSF biomarkers in AD?

The indications for the CSF examination , in general, did not change since the last Brazilian Academy of Neurology consensus^92^. CSF examination is thus indicated in the investigation of presenile dementia (before 65 years) and in cases with atypical clinical presentation or course, communicating hydrocephalus, and any evidence or suspicion of inflammatory, infectious, or prion disease of the central nervous system.

If the entire diagnostic process involving anamnesis, cognitive assessment, general examination, and neuroimaging studies, the etiology of the dementia syndrome remains doubtful, CSF biomarkers may be helpful. This uncertainty commonly arises in the differential diagnosis of atypical AD presentations with other dementias, such as the behavioral variant of FTD or agrammatic and semantic PPA.

CSF biomarkers can also be used in the workup for cognitive disorders to predict dementia in oligosymptomatic cases, where the identification of the AD ‘pathological signature’ in MCI allows inferring the underlying etiology of the disease with high accuracy.

The development of potentially disease-modifying drugs, such as anti-Aβ monoclonal antibodies, will promote CSF biomarker assessment, which is essential for indicating these medications.

### Biological diagnosis of AD through biomarkers

Diagnosis assisted by biomarkers allowed formulating recommendations for the biological diagnosis of AD by the A/T(N) classification^122^. According to this proposal, suspected cases of AD can be classified according to the positivity of biomarkers that allow inferring the underlying presence of one or more pathogenic processes characteristic of AD, where: “A” is the information obtained by biomarkers indicative of cerebral Aβ accumulation (i.e., Aβ1-42 reduction in the CSF or positivity in molecular imaging methods of amyloid detection); “T” is the positivity of biomarkers indicative of tau hyperphosphorylation (increased P-Tau in CSF or positivity in molecular imaging methods with tau-PET); and “N” indicates the occurrence of neurodegeneration by the increase of T-Tau levels in the CSF, hippocampal and/or temporoparietal atrophy in MRI, or loss of regional cerebral metabolic integrity, according to glucose uptake profile by [18F]FDG-PET^122^ ^),(^ ^123^. According to specific topographic patterns, such measurements are further related to the presence of degenerative process at an early stage, corresponding to the degree of cerebral atrophy and cognitive decline^115^ ^),(^ ^124^.

According to the 2018 NIA/AA recommendations, which included the AT(N) classification, the AD diagnostic spectrum requires evidence of accumulation of Aβ peptide and AD is defined by the combination of the positivity of this biomarker (A+) and biomarkers indicative of phosphorylated tau protein (T+). Positivity of biomarkers for cerebral amyloidosis (A+) and neurodegeneration (e.g., increase in CSF total tau) (N+) in the absence of P-tau increase (T-) markers concomitantly reflects AD pathological alteration and suspected non-AD pathological process. In short:

A -: not in the AD spectrum
A+: AD spectrum
A+/T+: AD
A+/T-/N+: AD + suspicion of another non-AD pathological process

NIA/AA authors^122^ advise that these recommendations apply only to research (observational and interventional) and should not be understood as guidelines or diagnostic criteria for clinical practice, considering that: (1) they do not consider clinical symptoms, signs, and functional impairment; and (2) 40% of cognitively normal older adults may present AD biomarkers and neuropathological alterations^125^, of which about 20% will never develop dementia, even in their nineties^126^.

### Pathological diagnosis of AD

The pathological diagnosis of AD is based on the presence of cortical atrophy, especially in the hippocampus and frontoparietal regions (associative areas), with marked neuronal loss and extracellular NPs and intraneuronal NFTs, which are the histopathological markers of AD that establish its definitive diagnosis. These markers are initially formed in the limbic system (hippocampus and entorhinal cortex), progressing to the association cortex, subcortical nuclei, and brainstem structures. Other neuropathological findings include neuronal loss in the pyramidal layers of the cerebral cortex and synaptic degeneration affecting associative limbic and cortical areas, starting from the hippocampus, with relative preservation of the primary areas (motor, somatosensory, and visual).

### Diagnosis of AD at different levels of health care

In Brazil, economic realities and access to health services are quite heterogeneous. This requires adapting the instruments and diagnostic approaches within the levels of care established in the Guidelines of the Unified Health System (Sistema Único de Saúde - SUS) (Primary, Secondary, and Tertiary Care).

Table 2 shows the suggested protocols for the evaluation and diagnosis of AD at each level of care:

**Table 2:** Suggested protocol for the diagnosis of AD at each level of health care.

Diagnosis in Primary Care	
Anamnesis	Ask the patient and their relative/caregiver about cognitive, neuropsychiatric, and behavioral symptoms. The interviews should be preferably conducted separately.
Clinical examination	Perform general physical examination looking for signs of systemic diseases and a complete neurological examination attentive to focal signs.
Laboratory tests	Perform laboratory tests to detect causes of secondary dementias and comorbidities that may contribute to the clinical picture.
Cognitive assessment	Applying a brief cognitive screening test, such as MoCA, BBRC, or CASI-S is suggested. BBRC stands out due to its easy application and high sensitivity, even for individuals with low schooling levels. Moreover, applying brief highly sensitive tasks, such as semantic verbal fluency (animals) and a word-learning test, which are highly accurate to detect dysfunction of the hippocampal system (amnesia), is recommended. Data on functionality can be addressed in the anamnesis or specific brief questionnaires, such as the Functional Activities Questionnaire (FAQ).
Structural neuroimaging	A brain CT is essential to rule out other causes of dementia (such as tumors, hydrocephalus, or cerebral infarctions) and to identify, within the limitations of the method, patterns of atrophy compatible with AD.
**Diagnosis in Secondary Care**	
Anamnesis	Ask the patient and their relative/caregiver about cognitive, neuropsychiatric, and behavioral symptoms.
Clinical examination	Perform general physical examination looking for signs of systemic diseases and a complete neurological examination attentive to focal signs.
Laboratory tests	Perform laboratory tests to detect causes of secondary dementias and comorbidities that may contribute to the clinical picture. If chronic meningitis is suspected, a lumbar puncture should be performed for CSF analysis.
Cognitive assessment	Using a brief test of cognitive screening or multifunctional battery of medium coverage is suggested, with at least one task to examine each cognitive domain; complement by applying a functional assessment tool, as described above.
Structural neuroimaging	Brain CT or MRI (preferably) is essential to rule out other causes of dementia and further investigate mesial structures, with visual scales or manual or automated volumetry.
Biomarker assessment	Using biomarkers is indicated in cases of diagnostic doubt between AD and other neurodegenerative dementias not in the amyloid spectrum, such as FTD, to correctly follow the guidelines for the use of AD approved medications, such as cholinesterase inhibitors and memantine. Other situations are referred to in biomarkers sections. Importantly, the future emergence and availability of potentially disease-modifying drugs will require evaluating all patients with mild AD as potential candidates for these treatments. Biomarkers can be requested from the secondary level of health care if they are reserved for selected cases and requested and interpreted by trained professionals.
**Diagnosis in Tertiary Care**	
Anamnesis	Conduct a detailed initial interview with the patient and their relative/caregiver.
Clinical examination	Perform a general physical examination looking for signs of systemic diseases and a complete neurological examination attentive to focal signs.
Laboratory tests	Perform laboratory tests to detect causes of secondary dementias and comorbidities that may contribute to the clinical picture and other tests if suspecting a relevant systemic disease. If chronic meningitis is suspected, a lumbar puncture should be performed for CSF analysis.
Cognitive assessment	A comprehensive neuropsychological evaluation using a cognitive screening test is suggested for applying instruments to further examine all cognitive domains and to assess functionality and neuropsychiatric disorders.
Structural neuroimaging	Brain MRI is essential to rule out other causes of dementia and further investigate the mesial temporal and other brain regions using visual scales or manual or automated volumetry.
Functional neuroimaging	FDG-PET or single-photon emission computerized tomography (SPECT) studies may show regional alterations in brain metabolism or changes in blood flow in cases of incipient and/or mild dementia, even in the absence of structural changes in neuroimaging.
Biomarker assessment	Using biomarkers is indicated in cases of diagnostic doubt between AD and other neurodegenerative dementias not in the amyloid spectrum, such as FTD, to correctly follow the guidelines for the use of AD approved medications, such as cholinesterase inhibitors and memantine. Other situations are referred to in biomarkers sections. Importantly, the future emergence and availability of potentially disease-modifying drugs will require evaluating all patients with mild AD as potential candidates for these treatments.

## FUTURE PROSPECTS

### Diagnosis of preclinical AD

The pathogenic process of AD begins many years before the first clinical manifestations of the disease, and the analysis of biomarkers indicates the presence of asymptomatic individuals^127^. Preclinical AD is therefore a long and silent stage of the disease that precedes the first cognitive alterations which will later lead to the diagnosis of mild cognitive impairment (MCI) due to AD. It corresponds to a window of opportunity to implement interventions for delaying (or, ideally, interrupting) the pathogenic process of AD^128^. The existence of interventions modifying the pathogenesis of AD, associated with the possibility of identifying the disease in the asymptomatic phase will represent an effective way to establish the prevention of dementia.

The diagnostic criteria for preclinical AD, restricted to the research context, were proposed to identify individuals at risk of AD in the asymptomatic phase^129^ ^)-(^ ^131^. Three evolutionary stages inherent to preclinical AD were proposed: the first shows isolated evidence of cerebral amyloidogenesis according to the positivity of Aβ biomarkers; the second shows evidence of ongoing neurodegenerative process according to CSF and/or brain imaging biomarkers; and the third, indicates very subtle cognitive or behavioral alterations, insufficient for the diagnosis of MCI^129^.

### Peripheral biomarkers of AD

Limitations for using these methods to better diagnose AD include the low availability and high cost of molecular image obtainment by PET and the need to perform lumbar puncture to obtain CSF samples. Developing new biomarkers in peripheral blood, with good diagnostic accuracy and predictive sensitivity, would thus significantly advance laboratory instrumentation in AD diagnosis. Moreover, determining plasma levels of Aβ, tau protein, and neurofilament light chain (NFL) -another neuronal cytoskeletal protein - with ultrasensitive methods could provide reliable estimates of cerebral amyloidogenesis and neurodegeneration in early stages of AD^121^ ^),(^ ^132^ ^),(^ ^133^. Plasma amyloid levels present a reliable correlation, measured by the relationship between peptides Aβ_1-42_ and Aβ_1-40_, and future positivity in amyloid PET^134^. Phosphorylated tau levels are present in other degenerative disorders but have been reported as elevated in plasma in individuals with AD, with the ^181^P-tau form showing greater specificity^133^ ^),(^ ^135^. The presence of NFL in CSF indicates nonspecific neuronal damage. Recent studies have shown a positive correlation between plasma and NFL cerebrospinal fluid levels, but only regarding neurodegeneration^136^. A recent meta-analysis showed that NFL levels in both CSF and plasma have high diagnostic sensitivity for AD and other neurodegenerative dementias^137^.

Other approaches using genomics, transcriptomics, metabolomics, lipidomics, and proteomics have been applied to generate different biomarkers for AD. One study showed that altered microRNAs resulting from the failure of the synaptic function are potential plasma biomarkers for AD^138^. A Brazilian study showed decreased levels of ADAM10 PPA-secretases in platelets, decreased PSEN1 levels in platelets and leukocytes, and lower bace1 (β-secretase) levels in leukocytes^139^.

### Implications of early diagnosis for disease-modifying therapies

The biomarker-based classification system proposed in 2018 indicates a broader concept of the pathological process in AD. However, clinical trials still face many challenges. Despite a growing understanding that clinical evaluation alone is limited for evaluating an intervention outcome, cognitive improvement measures are still the main outcomes in all clinical trials. Identifying the best molecular targets or a combination of them by developing better protocols to assess the results of interventions using biochemical, physiological, and neuropsychological measures as outcomes is essential to identify individuals in preclinical stages of AD and facilitate early therapeutic interventions. This is the premise of most efforts to find new therapies.
